# Alphavirus nsP2: A Multifunctional Regulator of Viral Replication and Promising Target for Anti‐Alphavirus Therapies

**DOI:** 10.1002/rmv.70030

**Published:** 2025-03-10

**Authors:** Sainan Wang, Suresh Mahalingam, Andres Merits

**Affiliations:** ^1^ Institute of Bioengineering University of Tartu Tartu Estonia; ^2^ Institute for Biomedicine and Glycomics Griffith University Gold Coast Australia; ^3^ Global Virus Network (GVN) Centre of Excellence in Arboviruses Griffith University Gold Coast Australia; ^4^ School of Pharmacy and Medical Sciences Griffith University Gold Coast Australia

**Keywords:** alphavirus, alphavirus replication, antiviral therapy, nsP2 inhibitor, superinfection exclusion

## Abstract

Alphaviruses are re‐emerging vector‐born pathogens that cause arthralgia or encephalitic diseases on a global scale. While a vaccine against chikungunya virus was recently approved, no vaccines currently exist for other alphaviruses, nor are there antiviral drugs for the treatment of alphavirus infections. Alphaviruses have positive‐strand RNA genomes, and their RNA replication is coordinated by activities of the multifunctional nonstructural protein 2 (nsP2), a helicase‐protease and a subunit of viral RNA replicase. We provide a comprehensive overview of nsP2 functions and inhibitors of its activities for their potential as effective antivirals. Furthermore, analysis of nsP2 activities suggests that it could be targeted to develop advanced live attenuated vaccines and strategies for controlling alphavirus transmission by mosquito vectors.

**Abbreviations**
Ala: alanine
AUD: alphavirus unique domain
BFV: Barmah Forest virus
BHK‐21: baby hamster kidney cell
cAMP‐PKA‐eEF2K: cyclic adenosine monophosphate ‐ protein kinase A—eukaryotic elongation factor‐2 kinase
CC_50_
: cytotoxic concentration 50
CHIKV: chikungunya virus
CPV‐1: cytopathic vacuoles type 1
de‐MARylation: de‐mono‐ADP‐ribosylation
ds: double‐stranded
EC_50_
: effective concentration 50
EEEV: Eastern equine encephalitis virus
eEF2: eukaryotic elongation factor 2
EILV: Eilat virus
FHL1: four‐and‐a‐half LIM domain protein 1
FRET: fluorescence resonance energy transfer
G3BPs: Ras‐GTPase activating SH3 domain‐binding proteins
Glu: glutamic acid
Gly: glycine
h.p.i.: hours post‐infection
hnRNP‐K: heterogeneous nuclear ribonucleoprotein K
HT‐Y2H: high‐throughput yeast two‐hybrid
IFN: interferon
JAK‐STAT: Janus kinase–signal transducer and activator of transcription
MAYV: Mayaro virus
MTL: methyltransferase‐like
NHC: β‐d‐N4‐hydroxycytidine
NLS: nuclear localization signal
ns: nonstructural
NSAIDs: non‐steroidal anti‐inflammatory drugs
nsP: nonstructural protein
nsP2h: nonstructural protein 2 helicase region
nsp2p: nonstructural protein 2 protease region
NTD: N‐terminal domain
NTPase: nucleoside triphosphatase
ONNV: o'nyong'nyong virus
ORF: open reading frame
RC: replication complex
RecA: RNA helicase core domain
RNAi: RNA interference
Rpb1: DNA‐dependent RNA polymerase II subunit 1
RRV: Ross River virus
RTPase: RNA triphosphatase
SAM: S‐adenosyl‐L‐methionine
SF1: superfamily 1
SFV: Semliki Forest virus
SG: subgenomic
SI: selectivity index (CC_50_/EC_50_)
SIE: superinfection exclusion
SINV: Sindbis virus
STAT1: signal transducer and activator of transcription 1
UBQLN4: ubiquitin 4
UPR: unfolded protein response
VEEV: Venezuelan equine encephalitis virus
WEEV: Western equine encephalitis virus

## 1 Introduction

Alphaviruses (genus *Alphavirus*, family Togaviridae) are enveloped viruses with positive‐strand RNA genomes. Currently, there are more than 30 recognized alphavirus species, most of which are transmitted between vertebrate hosts via invertebrate vectors, primarily mosquitoes, though ticks, lice and cliff swallow bugs can also play a role in transmission. Alphaviruses can infect humans, domestic animals such as pigs and equids, and wild mammals, including non‐human primates and rodents, as well as birds, reptiles, amphibians and fish [1, 2, 3, 4, 5]. Arthropod‐transmitted members of the genus are divided into eight phylogenetic complexes [1]. Human infections with arthritogenic alphaviruses, such as chikungunya virus (CHIKV), Sindbis virus (SINV), Ross River virus (RRV), Mayaro virus (MAYV) and o'nyong'nyong virus (ONNV), primarily result in fever and polyarthritis. Currently, CHIKV is the most medically important alphavirus, with approximately 480,000 human cases and 190 deaths reported in 2024. Its healthcare burden is further increased by its ability to cause chronic arthritic symptoms that can last months or even years in approximately 30%–60% of patients [6, 7, 8, 9]. As their names suggest, Venezuelan, Eastern and Western equine encephalitis viruses (VEEV, EEEV and WEEV) cause encephalitis in humans and domestic animals [10, 11]. The fatality rate for symptomatic human infections with EEEV reaches 30%. Table 1 lists medically relevant alphaviruses, their geographical distribution, vectors, reservoir hosts and disease symptoms in humans.

**TABLE 1:** Vectors, hosts and symptoms of pathogenic alphaviruses.

Alphavirus	Confirmed or putative mosquito vectors	Confirmed or putative reservoir hosts	Incubation period	Symptoms in humans	Occurrence
Chikungunya virus (CHIKV)	*Aedes aegypti*; *Aedes albopictus* [12]	Nonhuman primates [13]	2–12 days	Main symptoms include fever, myalgia, polyarthralgia, rash, headache and chronic symptoms in up to 80% of patients. Other symptoms include nausea, vomiting, tenosynovitis conjunctivitis, occasional bleeding gums, and epistaxis [14, 15]. Rare atypical symptoms include encephalitis, meningitis, and Guillain‐Barré syndrome [16, 17].	Americas, Africa, Asia, Europe and the Indian and Pacific Oceans
o'nyong'nyong virus (ONNV)	*Anopheles funestus*; *Anopheles gambiae* [18]	Unknown	8 days	Similar to CHIKV in major symptoms (usually milder) with the addition of 71% pruritus and 45% cervical lymphadenitis [19].	Africa
Barmah Forest virus (BFV)	*Culex annulirostris* [20, 21]	Not established, but it is likely that macropods and marsupials are the hosts	7–9 days	Fever, myalgia, rash, headache, and lethargy [22, 23].	Australia
Mayaro virus (MAYV)	*Haemagogus janthinomys* [24]; *A. aegypti* [25]	Nonhuman primates [26]	7–12 days	Main symptoms are similar to CHIKV (mostly milder); however, about 50% of patients develop oedema, retroocular pain, and 10%–20% of patients have swollen lymph nodes and diarrhoea [27].	Latin America, Caribbean
Ross River virus (RRV)	*Culex annulirostris*; *Aedes vigilax* [28]	Marsupials [29]	3–21 days	Fever, myalgia, rash, headache, photophobia, and rarely encephalitis [30].	Australia, new Guinea, Fiji, the Solomon Islands, American Samoa, South Pacific islands
Sindbis virus (SINV)	*Culex pipiens*; *Culex univittatus* [31]	Thrush family (Turdidae) [32]	2–10 days	Fever, rash, myalgia, arthralgia [33, 34].	Europe, Africa, Australia, Asia, Philippines
Venezuelan equine encephalitis virus (VEEV)	*Ochlerotatus taeniorhynchus*; *Culex melanoconion*; mammalophilic mosquitoes [35, 36]	Equines and sylvatic rodent [37]	2–5 days	Usually mild flu‐like illness with rarely fatal (less than 1%) encephalitis [36, 38].	Latin America and the USA
Western equine encephalitis virus (WEEV)	*Culex tarsalis*; *Aedes albifasciatus* [39]	Birds and lagomorphs [40]	2–7 days	From mild febrile flu‐like illness to severe or fatal (3%–7%) encephalitis [36].	The USA, Canada and the southern cone region of South America
Eastern equine encephalitis virus (EEEV)	*Culiseta melanura;* [41] *Culex erraticus* [42]	Passeriformes birds [41]	4–10 days	Similar to WEEV, however, the fatality can be 50%–70% [36].	Mainly in the eastern coastal areas of the USA and Canada, the Caribbean and Argentina

Alphaviruses are known to cause periodic large‐scale outbreaks, such as the CHIKV epidemic in the Indian Ocean region from 2004 to 2011, with millions of human cases [43, 44]. Increased international travel, expanded distribution of mosquito vectors, and adaptive changes of alphaviruses to the new vectors [12, 45] have made these viruses a potential ‘Disease X’ threat. Currently, only one live‐attenuated vaccine has been approved to prevent CHIKV infection [46]; however, there are no vaccines for other alphaviruses or specific antiviral drugs available. Treatment of alphavirus infections primarily aims to relieve disease symptoms and involves the use of analgesics and/or NSAIDs (non‐steroidal anti‐inflammatory drugs). Understanding the replication mechanisms of alphaviruses is crucial for developing effective countermeasures.

**FIGURE 1:**
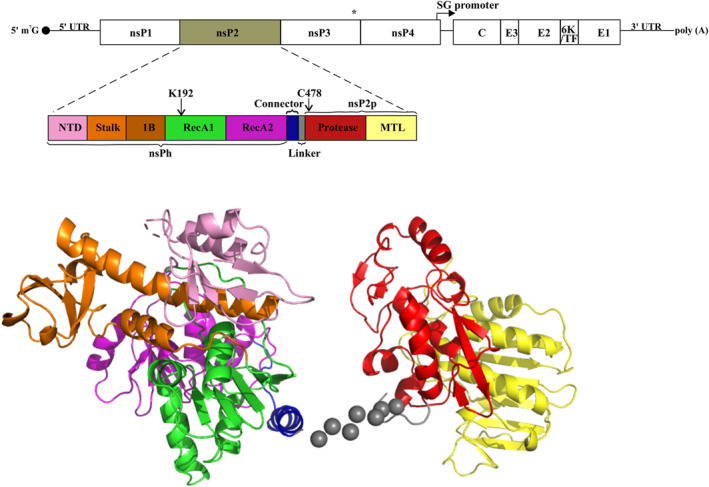
The organisation of the alphavirus genome and domain organisation of nsP2 protein of CHIKV. * designates an opal stop codon that is present at the end of nsP3 region of the majority of alphaviruses. nsP2 consists of nsP2h and nsP2p regions connected by flexible linker; the sub‐domains of nsP2h and nsP2p are colour‐coded. The 3D structure of CHIKV nsP2 is kindly provided by Yee–Song Law [49]. NTD (residues 1–26), N‐terminal domain; Stalk (residues 27–109); 1B (residues 110–174), superfamily 1 accessory domain 1B; RecA1 (residues 175–308) and RecA2 (residues 309–439), conserved Rec‐A‐like domains; connector and linker (residues 440–464); protease (residues 465–604) and MTL (residues 605–798), S‐adenosyl‐L‐methionine (SAM)‐dependent RNA methyltransferase‐like domains are shown. Arrows point to the active site residues of NTPase/helicase (K192) and protease (C478).

Alphavirus RNA genome has a 5′ methylguanylate cap and a 3′ polyadenylate tail, with two open reading frames (ORFs) [47]. The first ORF encodes the nonstructural (ns) polyproteins P123 (90%) and P1234 (10%) [48], which are precursors to 4 ns proteins (nsP1‐4). The second ORF encodes structural polyproteins, translated from the subgenomic (SG) RNA produced in infected cells (Figure 1) [10]. RNA replication of alphaviruses is highly regulated by the ns polyprotein processing; both processing intermediates and mature nsPs are important for RNA replication. nsP1 is the membrane anchor of the replication complex (RC) [50, 51] and is essential for capping newly synthesised genomic and SG RNAs [52, 53]. nsP2 is a cysteine protease that orchestrates P1234 polyprotein processing [54, 55] and has RNA helicase, nucleoside triphosphatase (NTPase) and RNA triphosphatase (RTPase) activities [56, 57, 58, 59]. nsP3 acts as an interaction hub for host factors and is crucial for negative‐strand and SG RNA synthesis [60, 61, 62, 63, 64, 65, 66]. It assembles into a crown‐like structure associated with RC and forms bundles of tubular structures in infected cells [67]. nsP4 is the RNA‐dependent RNA polymerase and terminal adenylyltransferase; it is also shown to counteract antiviral effects of the unfolded protein response (UPR) [68, 69, 70, 71].

## 2 Organisation of nsP2 and Its Enzymatic Activities

In this review, we focus on alphavirus nsP2, a ∼90 kDa protein with multiple interconnected functions that play a central role in RNA replication. For arthritogenic alphaviruses, nsP2 also counteracts the host antiviral response by shutting down cellular transcription and acting as a critical determinant of viral pathogenesis [72, 73, 74]. Reflecting its multiple functions, nsP2 has a complex structure: its N‐terminal RNA helicase region (nsP2h) is connected to C‐terminal protease region (nsP2p) via a short flexible linker [49]. nsP2h belongs to a superfamily 1 (SF1) of helicases and consists of N‐terminal domain (NTD), stalk, superfamily 1 accessory domain 1B and two similar Rec‐A‐like domains (RecA1 and RecA2). The conserved helicase motifs establish polar contacts with the RNA backbone. The two aromatic residues of domain 1B (Y161 and F164) form stacking interactions with RNA bases and are crucial for CHIKV viability [59]. A recombinant nsP2h has NTPase and RTPase activities, but the presence of nsP2p increases nsP2h's NTPase activity by 5–7 fold, and only the full‐length nsP2 demonstrates RNA helicase activity [75]. The nsP2p region consists of two domains. A papain‐like cysteine protease domain contains a catalytic dyad formed by cysteine and histidine residues [55, 76, 77]. A S‐adenosyl‐L‐methionine (SAM)‐dependent RNA methyltransferase‐like (MTL) domain that lacks RNA methyltransferase activity (Figure 1). This domain contains numerous positively charged amino acid residues, binds RNA at sequence‐independent manner and regulates enzymatic activities of nsP2 [78, 79, 80]. nsP2 recognises and cleaves three cleavage sites within the P1234 polyprotein: the sites between nsP1 and nsP2 (1/2 site), nsP2 and nsP3 (2/3 site) and nsP3 and nsP4 (3/4 site). In cell‐free reaction nsP2p processes 3/4 site more efficiently than 1/2 site. Studies have shown that the amino acid residues at positions P4–P1' (nomenclature of Schechter and Berger is used here and below) in the 3/4 site play an essential role in cleavage efficiencies [81]. However, apart from the invariable P2 Gly residue, the P4–P1′ sequences typically differ across sites, partially accounting for varying cleavage efficiencies. These differences alone, however, do not fully explain the order of cleavage events, suggesting the existence of additional recognition mechanisms [82]. This is particularly evident for the 2/3 site, which can be processed only by full‐length nsP2. Furthermore, its cleavage requires the presence of the first domain (macro‐domain) of nsP3, which functions in precisely positioning the 2/3 site within nsP2's protease active site in the substrate [83, 84].

Taken together, the two seemingly different regions of nsP2 (nsP2h and nsP2p) are functionally coupled. This also reflects the crucial role of the linker connecting these regions, as deletion of 3 or 5 linker residues is lethal for CHIKV [49]. This functional coupling also extends to nsP3, which was found to regulate nsP2p activity directly. Specifically, nsP3's macro‐domain mediated de‐mono‐ADP‐ribosylation (de‐MARylation) activity is required to remove MARylation (cellular modification inactivating protease activity of nsP2) from nsP2, thereby reactivating its proteolytic function [85]. Thus, the various functions of nsP2 and its interactions with other nsPs must be well coordinated to support its essential role in viral RNA replication. However, the precise mechanism(s) underlying these coordinated activities remain unclear.

## 3 nsP2 Regulates the Formation of Alphavirus RCs

**FIGURE 2:**
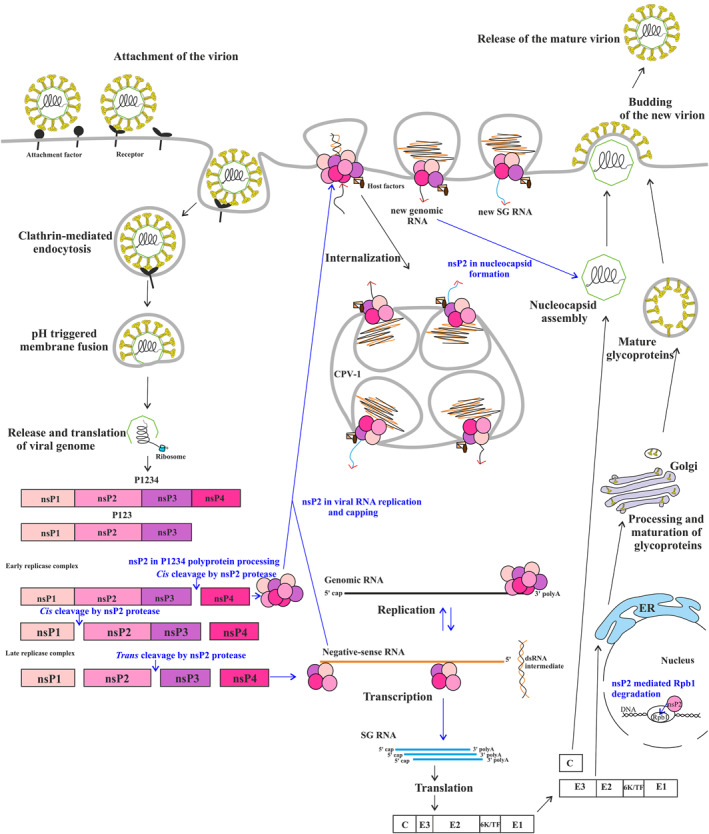
Alphavirus infection cycle. Infection is initiated by the binding of virions to cellular receptor(s) and their entry by clathrin‐dependent endocytosis. At low pH, the virion envelope fuses with endosomal membrane, releasing the nucleocapsid into the cytoplasm. The genomic RNA is then uncoated and translated. Step‐by‐step processing of P1234 polyprotein results in the formation of an early replicase complex and its conversion to late replicase complex; this co‐insides with the formation of spherules at the plasma membrane. For SFV and RRV, spherules are internalized and, at later stages of infection, localise on the internal surface of modified endosomes and lysosomes, forming so‐called cytopathic vacuoles (CPV‐1) [86]. The dsRNA molecule localises inside of spherule and is used by the late replicase to produce new genomic and SG RNAs. The SG RNA serves as a mRNA for translation of structural proteins required for assembly and release of progeny virions. Spheres indicate ns‐proteins included in the active replicase complexes (stoichiometry and exact localization of replicase subunits is shown on Figure 3). Blue arrows indicate the key activities of nsP2: processing of P1234 polyprotein, assisting of viral RNA replication, participation in the capping of positive strand RNAs, involvement in formation of nucleocapsids, and (for arithrogenic alphaviruses) suppression of cellular transcription by degradation of Rpb1. Red arrowheads indicate the direction of RNA transport in the neck of spherule.

Alphavirus genome replication is initiated and coordinated through the processing of P1234 polyprotein (Figure 2) [54]. First, P1234 polyprotein is rapidly cleaved at the 3/4 site. In infected cells, the cleavage occurs in cis; however, as the site is easily accessible for nsP2, cleavage *in trans* is also possible. Regardless of the mode of cleavage, the release of nsP4 results in the formation of a short‐lived early replicase complex (P123 polyprotein + nsP4) that synthesises negative‐strand RNA using the genomic RNA as a template, thereby forming dsRNA. The physical structure and precise composition of this early replicase are not yet known. In Semliki Forest virus (SFV) and SINV, the 1/2 site is cleaved slowly and exclusively, or almost exclusively, in cis [87]; acceleration of its processing results in attenuated or non‐viable viruses [88], suggesting that this cleavage is purposefully delayed. The resulting nsP1 + P23 + nsP4 complex has a very short half‐life because the formed P23 polyprotein is rapidly cleaved into nsP2 and nsP3. This cleavage occurs *in trans*, as in the P23 polyprotein, the 2/3 site is over 40 Å from the active site of nsP2p and cannot be cleaved by the same molecule [80]. The late RNA replicase (nsP1 + nsP2 + nsP3 + nsP4) then uses the negative strand of dsRNA as a template to synthesise large amounts of genomic and SG RNAs. This synthesis occurs in a bulb‐like membrane invaginations, or spherules, approximately 50 nm in diameter, which initially form at the plasma membrane of infected cells (Figure 2) [86, 89]. In SFV‐infected cells, spherules are subsequently internalized using phosphatidylinositol‐3‐kinase‐, actin‐ and microtubule‐dependent transport and finally localise on the internal surface of modified endosomes and lysosomes forming so‐called cytopathic vacuoles (CPV‐1) [86]. Disruption of this endocytic process by treatment with blebbistatin, wortmannin, or other inhibitors has little to no impact on the viral RNA synthesis [86, 90]. In contrast, in CHIKV‐infected cells, the majority of spherules remain associated with the plasma membrane. Different phenotypes of SFV and CHIKV are due to differences in activation of phosphatidylinositol‐3‐kinase‐Akt‐mTor pathway mediated by nsP3 proteins of these viruses [91]. Furthermore, size of the SFV spherules is uniformal and determined by the length of replicating viral RNA [92]. However, in CHIKV‐infected human cells, spherules that remain associated with the plasma membrane are unexpectedly heterogeneous [92]. The large diameter of these compartments, which remain dynamically active in viral RNA synthesis, suggests a continuous growth of these organelles beyond the replication of a single RNA genome [93]. Do these differences have an impact on other properties of these viruses remains currently unknown.

Thus, all key events in alphavirus RNA synthesis depend on the protease activity of nsP2. However, only cleavage at the 3/4 site is absolutely required for RNA replication. In contrast, blocking processing at the 2/3 site or at both the 1/2 and 2/3 sites is not lethal; SINV mutants with two blocked cleavage sites can still replicate in interferon‐defective cells to levels comparable to the wild‐type virus [94, 95].

## 4 nsP2: A Part of Core‐Structure of Alphavirus RNA Replicase

**FIGURE 3:**
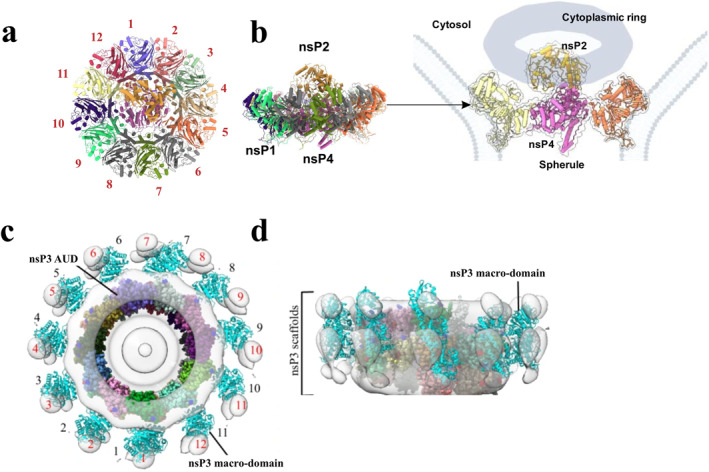
Structure of alphavirus replicase complex. (a), a top view, and (b), a side view of the replicase core reconstructed from recombinant nsP1, nsP2, and nsP4 proteins. In the core structure of replicase complex one molecule of nsP4 sits within the central pore of the dodecameric ring formed by nsP1. The nsP4 interacts with the NTD and Stalk sub‐domains of nsP2h. Image of the complex located in the neck of spherule is shown on right; the nsP2p is considered to be confined to a cytoplasmic ring structure. (c), a top view, and (d), a side view of the cytoplasmic ring composed of 11 nsP3 molecules. AUD, alphavirus unique domain of nsP3. Images are adapted from Tan et al., 2022 (a, b) and Kril et al., 2024 (c, d) [51, 67].

The core of alphavirus late replicase consist of a ring structure formed by 12 molecules of nsP1 [96, 97], with a single nsP4 molecule situated in the central pore of the nsP1 ring and 1 nsP2 molecule positioned on the cytosolic side of the complex (Figure 3). Within this complex, the NTD and stalk sub‐domains of nsP2h interact with the palm sub‐domain of nsP4. Notably, nsP1 and nsP4 complexes can be formed from purified recombinant proteins [51]. Adding recombinant nsP2 to these complexes increases their RNA polymerase activity and enables the terminal adenylyltransferase activity, indicating cooperative interactions between nsP4 and nsP2.

Only a fraction of RNA replicase cores formed in infected cells are associated with spherules, and localise at the neck region of these structures (Figure 3). While the precise localization of nsP2p remains unclear, it is thought to be confined to an amorphous cytoplasmic ring structure associated with spherules [51]. Cytoplasmic ring, composed of 11 subunits of nsP3, incorporates host factors such as G3BPs (Ras‐GTPase activating SH3 domain‐binding proteins) and FHL1 (four‐and‐a‐half LIM domain protein 1) [67]. Interestingly, majority of replicase cores are not associated with spherules, lack cytoplasmic ring structures [51], and are presumably not involved in the viral RNA synthesis. Their potential functions remain unknown. What is known is that nsP2 also plays a role in regulating the formation of RCs; excessive nsP2 levels lead to the cleavage of P1234 polyprotein into P12 and P34, which cannot form functional RCs [98].

One of the least understood aspects of alphavirus RNA replication is the mechanisms of the template RNA recognition and interactions between replicase proteins and genomic RNA (and dsRNA). Although nsP2 can bind RNA, evidence for sequence‐specific binding is indirect. Changes in the 5′ *cis*‐acting RNA elements of the viral genome result in the compensatory mutations in nsP2h [99, 100], and temperature‐sensitive mutations in nsP2h have been shown to influence efficiency of SG RNA synthesis [101, 102]. As alphavirus nsP2 helicase translocates along ssRNA in a 5′ to 3′ direction [103], it could be assumed that nsP2 guides the 3′ end of genomic RNA into the spherule via the putative RNA channel formed by nsP1 and nsP4. However, recombinant nsP2 possesses low RNA helicase activity and very limited processivity. Interestingly, despite its designation as a nonstructural protein, nsP2 of SINV has been shown to be present in its virions [104]. While the exact reason for nsP2's inclusion into virions remains unclear, it is hypothesised that nsP2, the viral genome, capsid proteins and host factors such as G3BPs are interconnected [105], suggesting a link between alphavirus RNA synthesis and its encapsidation. Supporting this, mutations introduced into nsP2 of SFV result in the potential compensatory changes in the capsid protein [88], and mutations introduced into capsid protein of VEEV were shown to cause compensatory mutations in nsP2 [106].

## 5 nsP2 Interacts With Host Proteins, Triggers Host Cell Shutoff and Enables Immune Evasion

nsP2 plays a critical role in alphavirus‐host interactions. In infected cells, numerous host proteins are pulled down via nsP2 [107]. However, many of these proteins interact with RCs of alphavirus and not necessarily with nsP2 itself as they can also be pulled‐down via nsP3 [108, 109]. nsP2 can also be found outside of the RCs or membrane‐bound nsP1:nsP4:nsP2 complexes and can directly interact with host proteins. High‐throughput yeast two‐hybrid (HT‐Y2H) assays identified heterogeneous nuclear ribonucleoprotein K (hnRNP‐K) and ubiquitin 4 (UBQLN4) as critical interactors of CHIKV nsP2, essential for viral replication. The interaction between hnRNP‐K and nsP2 has also been demonstrated for SINV [108, 110].

While free nsP2 localises diffusely in the cytoplasm or binds to ribosomes [107, 111, 112], a significant fraction of it accumulates in the nucleus [113, 114, 115]. In SFV‐infected vertebrate cells, approximately 50% of nsP2 resides in the nucleus, driven by nuclear localization signals (NLSs) [115, 116, 117]. For SFV, mutations in these NLSs prevent nuclear localization of nsP2, reducing RNA replication, cytotoxicity [118], and neurovirulence in mice [72]. However, for SINV and CHIKV, mutations in putative NLSs have little or no impact on the nuclear localization of nsP2 [119, 120].

Although the precise mechanism of nuclear transport remains controversial, nuclear accumulation of nsP2 is clearly critical for arithrogenic alphaviruses [74, 113]. In vertebrate cells, alphavirus infection induces host cell shutoff and prominent cytopathic effects [121]. For arithrogenic alphaviruses, these effects are mediated by nsP2. Mutations in the MTL domain of nsP2 reduce cytopathicity, enabling persistent replication [120, 122, 123]. The absence of host cell shutoff increases interferon (IFN) production, thereby inhibiting virus replication and spread in IFN‐competent cell cultures [113]. The nsP2 of SINV, SFV, CHIKV and MAYV inhibits host cell transcription by inducing the degradation of the catalytic subunit of DNA‐dependent RNA polymerase II (Rpb1) [73, 124]. This process is mediated by nsP2‐induced Rpb1 ubiquitination rather than nsP2's protease activity [123] and depends on the integrity of the helicase and S‐adenosylmethionine (SAM)‐dependent methyltransferase‐like domains. nsP2 induced transcriptional shutoff occurs before other virus‐induced changes, such as apoptosis, autophagy and inhibition of STAT1 phosphorylation, thereby allowing the virus to evade innate immune responses [73]. Functions of nsP2 in suppression of antiviral immune response are, however, not limited to the general shutoff of host cell transcription. For example, CHIKV nsP2 can specifically inhibit the IFN‐stimulated Janus kinase–signal transducer and activator of transcription (JAK‐STAT) signalling pathway [125]. These effects are, at least to an extent, also controlled by a negative feedback loop. For example, CHIKV nsP2 interacts with the human autophagy receptor NDP52. This interaction limits the amount of nsP2 in the nucleus, which reduces host shutoff and cytopathic effects and prolongs CHIKV replication in human cells [126]. NTPase activity of nsP2 also contributes to the translational shutoff of host cells through the cAMP‐PKA‐eEF2K signalling pathway [127]. Interestingly, for encephalitic alphaviruses such as VEEV and EEEV, transcriptional shutoff is mediated by the capsid protein [128], while nsP2 of VEEV plays a crucial role in the shutoff of host cell translation [129].

In summary, nsP2 plays a central role in various stages of alphavirus infection including RNA replication and packaging, with its multifunctional nature influencing replication fidelity, template recognition, and the coordination of RNA synthesis with viral assembly. Furthermore, it interacts with other viral and host components and has an important role for virus‐host interactions. The key activities of nsP2 of alphaviruses are summarised in Table 2.

**TABLE 2:** Main functions of alphavirus nsP2.

Activity of alphavirus nsP2	Functions	Importance
RNA helicase	Unwinding dsRNAs [56, 59, 75].	Crucial for viral RNA replication [59].
Nucleoside triphosphatase (NTPase)	Hydrolysis of NTPs [57].	Energy source of RNA helicase, important for viral RNA replication, contributes to the host cell translational shutoff [127].
RNA triphosphatase (RTPase)	Removal of *γ*‐phosphate from the 5′ end of genomic and subgenomic RNAs [58].	Crucial for viral RNA capping, essential for protection of viral RNAs from degradation and recognition by the host immune system [58].
Cysteine protease	Processing of non‐structural polyproteins [55].	Regulation of the replicase complex formation and viral RNA replication [55, 90].
Non‐enzymatic activities	Degradation of cellular DNA‐dependent RNA polymerase II (Rpb1) [73, 124].	Shut‐off of host cell transcription. Blocking of antiviral responses [73].
	Translocation to the nucleus and nucleolus of vertebrate cells [113, 114, 115].	Influencing ‐cytotoxicity and virulence of SFV [118].
	Interacting with components of RC, capsid, and host proteins [51, 105, 126].	Important for viral RNA replication and encapsidation of genomic RNA [51, 105, 108].
	Inhibition of IFN‐stimulated Janus kinase–signal transducer and activator of transcription (JAK‐STAT) signalling pathway [125].	Important in viral pathogenesis [125].

## 6 nsP2 as a Target for Antiviral Compounds

Alphaviruses cause acute infections and can lead to the development of chronic arthritic symptoms. The mechanism behind chronic disease caused by CHIKV infection remains poorly understood; however, studies suggest that macrophages may harbour persistently replicating virus, contributing to debilitating arthropathy [130]. Compounds targeting viral proteases are used clinically to treat both acute diseases, such as COVID‐19 with nirmatrelvir and chronic diseases, such as human immunodeficiency virus type 1 infection with lopinavir/ritonavir, darunavir, and others or hepatitis C with simeprevir, grazoprevir, and others [131, 132, 133, 134]. Alphavirus nsP2, which is crucial for RC formation and functioning as well as for suppression of the antiviral responses (Table 2), is, therefore, a promising target for antiviral drug development. However, the complex structure and interconnected functions of nsP2 have made it a challenging target.

To date, the majority of inhibitors of alphavirus nsP2 target its protease activity. This is partly due to the availability of high‐resolution 3D‐structures of nsP2p from several alphaviruses, including CHIKV (3TRK) and VEEV (2HWK) [78, 79], which facilitate structure‐based in silico drug design approaches [135, 136, 137, 138, 139]. The known 3D structures can also be used to predict the structures of proteases from other alphaviruses, expanding the list of potential targets. For example, Bassetto and colleagues utilised the structure of VEEV nsP2p to predict the 3D structure of CHIKV nsP2p, resulting in a model that closely resembles the actual 3D structure of CHIKV nsP2p, which became available several years later. The virtual screening identified hits that were shown to inhibit CHIKV replication in vitro with an effective concentration of 50 (EC_50_) of 5 μM [140]. A combination of in silico screening and a cell culture virus‐yield reduction assay was also employed to develop inhibitors of VEEV nsP2 protease [141]. However, this method lacks biological validation for target specificity, and later studies revealed that the compound identified by Bassetto and colleagues did not inhibit CHIKV nsP2 protease in a cell‐free assay [142]. To address this, an improved method was developed that integrates in silico screening, cell‐free protease assay using recombinant nsP2p and cell‐based virus‐yield reduction assay [142].

The cell‐free protease assay is based on the ability of nsP2p, a shorter and more easily expressed and purified region of the nsP2, to cleave *in trans* substrates corresponding to 1/2 or 3/4 sites in P1234 polyprotein. Due to the higher cleavage efficiency at the 3/4 site, a corresponding cleavable linker is used in both recombinant proteins and synthetic peptide‐based substrates. In the latter case, fluorescence resonance energy transfer (FRET) is used to detect processing [75, 142, 143, 144], enabling the use of high‐throughput screening (HTS) assays [141, 145, 146, 147, 148, 149]. Compounds reported to inhibit nsP2 are summarized in Table 3. However, it is important to note that results from cell‐free and cell‐based assays often differ. Some compounds that do not inhibit nsP2 protease activity in cell‐free assays have been shown to inhibit virus infection, while others that effectively inhibit nsP2 protease activity demonstrate little or no antiviral effects [142, 155, 159]. Such discrepancies are hardly surprising, as cell‐free assays do not account for the ability of compounds to penetrate the plasma membrane or their stability within cells. Furthermore, in the processing cascade leading to RC formation, both the 1/2 and 3/4 sites in P1234 polyprotein are cleaved in cis, and therefore, the requirements for their processing are likely different from those of substrates used in cell‐free reactions. In addition, as described above, the cleavage site requirements of nsP2 are complex. For instance, the 2/3 site cannot be cleaved by nsP2p alone and requires the presence of the nsP3 macro‐domain in the substrate. Finally, in infected cells, the consequences of lack of cleavage at different processing sites are drastically different: only cleavage at the 3/4 site is mandatory, whereas a slowdown in the cleavage of the 1/2 site (and possibly the 2/3 site) may even positively impact virus RNA replication. This latter property raises concerns regarding the sub‐optimal efficiency of nsP2 inhibitors, which, until recently, have demonstrated activities only in the micromolar range (Table 3). An important breakthrough was achieved in 2024 with the development of the first CHIKV nsP2 protease inhibitor with an EC_50_ of approximately 10 nM, using a covalent fragment‐based screening approach [146]. This achievement underscores the potential for clinically applicable nsP2 protease inhibitors and highlights the need for improved screening methods and advanced cell‐based assays (compatible with HTS) to identify and characterize these inhibitors.

**TABLE 3:** Compounds targeting alphavirus nsP2 protease.

Compound	Structure	Targeted virus	EC_50_ (μM)	CC_50_ (μM)	Selectivity index (SI = CC_50_/EC_50_)	Cell line	Ref.
(E)‐3‐(4‐tert‐butyl phenyl)‐N‐[(E)‐(3,4‐diethoxyphenyl) methylideneamino] prop‐2‐enamide (compound 25)	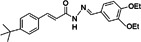	CHIKV	3.2 ± 1.8	101 ± 50	32	Vero	[140, 150, 151]
5‐chloro‐N‐[4‐[C‐methyl‐N‐[(2‐phenylcyclopropanecarbonyl) amino] carbonimidoyl] phenyl] thiophene‐2‐carboxamide (compound 8)	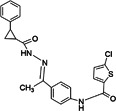	CHIKV	1.5	> 200	> 133.3	BHK21	[142]
ID1452‐2	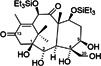	CHIKV	31	> 31	Not specified	HEK293	[152]
Novobiocin	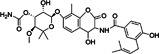	CHIKV	20	Not specified	Not specified	Vero	[153]
Telmisartan	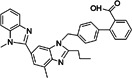	CHIKV	45	Not specified	Not specified	Vero	[153]
MBZM‐N‐IBT	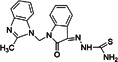	CHIKV	38.68	> 800	> 21	Vero	[154]
1,3‐thiazolidin‐4‐one (compound 7)	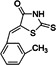	CHIKV	0.42	> 100	> 238	Vero	[155]
Peptidomimetic 3a	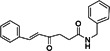	CHIKV	8.76	Not specified	Not specified	Vero	[156]
β‐Aminomethyl vinyl sulfone RA‐0002034	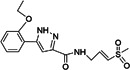	CHIKV	0.01	> 10 µM	> 1000	MRC5	[146]
LMQ330	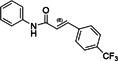	CHIKV	5.2 ± 0.52	165.3 ± 2.7	31.78	Vero	[149]
NCGC00488909‐01	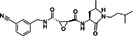	VEEV	1.76	> 50	> 28	A549	[141]
Vinyl sulfone‐based inhibitor (compound 11)	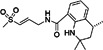	VEEV	2.4	30	12	HeLa	[157]
CA‐074	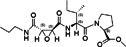	VEEV	Not specified	Not specified	Not specified	HEK293 vero A549	[158]

Compounds targeting other nsP2 functions are rare. No inhibitors of nsP2 RNA helicase, NTPase, or RNA triphosphatase activities have been reported. However, with the published structures of nsP2h and its complex with nsP4 [51, 160], the in silico design and cell‐based analysis of such compounds has become feasible. A cell‐based *trans*‐reporter assay, measuring the induction of luciferase gene expression by an artificial transcription factor, has been developed and used to identify compounds that block nsP2‐induced transcriptional shutoff and inhibit CHIKV replication [108, 152]. The use of mutations in nsP2 that permit persistent replication of alphavirus replicons (modified genomes in which ORF2 is replaced with a sequence encoding a reporter and/or selection marker) has led to the development of stable replicon cell lines. These cell lines serve as valuable tools for screening antiviral compounds targeting the functions of ns proteins. Stable cell lines harbouring noncytopathic CHIKV replicons have been used for antiviral screening, leading to the identification of *β*‐d‐N4‐hydroxycytidine (NHC), as well as 5,7‐dihydroxyflavones apigenin, chrysin, naringenin and silybin. Some of these compounds, among other mechanisms, may also target nsP2 functions and have been shown to suppress CHIKV replication [161, 162].

Alphavirus RNA replication is error‐prone; hence these viruses are expected to develop resistance against directly acting antivirals, including nsP2 inhibitors. As truly active nsP2 inhibitors are just starting to emerge, no data concerning virus resistance to such compounds is available. However, the resistance‐associated mutations have been used to identify nsP2 as the target of quinazolinone compound CID15997213, an inhibitor of VEEV and WEEV, as well as radicicol, a heat shock protein 90 (Hsp90) inhibitor that effectively suppresses CHIKV replication [163, 164]. Resistance‐associated mutations in nsP2, along with those in nsP4, were also obtained during the passaging of VEEV in the presence of benzamidine ML336 [165].Furthermore, mutations both in nsP2 and nsP4 emerged in response to treatment of CHIKV infected cells with mutagenic agent ribavirin [144] or with 4′‐fluorouridine, a chain terminator targeting nsP4 [166]. In both cases, it was observed that, on their own, mutations in nsP2 had little impact; however, they did slightly increase the impact of mutations located in nsP4. A similar situation was observed with the CHVB series, a novel class of CHIKV inhibitors that target the viral capping machinery. In this case, mutations in nsP1 were coupled with mutation located in the MTL domain of nsP2. Though the mutations in nsP1 were necessary and sufficient to achieve the resistance, it was suggested that mutation in nsP2 has some impact, possibly due to the interaction of MTL with nsP1 during the viral RNA capping [167]. Combinations or resistance‐associated mutations in nsP1 and nsP2 or in nsP4 and nsP2 may also reflect the molecular architecture of the alphavirus replicase core (Figure 3), where these proteins interact with each other and function in a coordinated manner.

It should also be taken into account that alphavirus nsP2 is a multifunctional fine‐tune instrument involved in many activities, from viral replication to shutoff of hosts’ antiviral responses. Therefore, it is also reasonable to expect that mutations, allowing resistants to nsP2 inhibitors, likely also affect other important functions of the protein. Thus, the resistance to nsP2 inhibitors is likely associated with high fitness costs. This may generate high genetic barriers for resistance, making the development of nsP2 inhibitors especially attractive.

## 7 nsP2 Can be Exploited for Alphavirus Attenuation and as a Potential Tool for Vector Control

The critical role of nsP2 in the host‐cell shutoff, counteraction of the IFN response, and the pathogenesis of alphavirus infections offers numerous opportunities to develop advanced alphavirus‐based gene expression systems, including improved self‐replicating RNAs and rationally attenuated viruses. These applications require precise knowledge of nsP2 functions. For example, selecting alphaviruses incapable of inducing transcriptional shutoff led to the identification of a short, highly variable peptide loop (VLoop) in nsP2 of SINV and CHIKV. Mutations in the VLoop enable the corresponding viruses to establish persistent, noncytopathic replication in mammalian cells [168] and attenuate CHIKV replication in vivo [169]. In another study, a mutation affecting the P4 residue of the 1/2 cleavage site in CHIKV, paired with a mutation in the corresponding S4 residue of substrate‐binding pocket of nsP2p [170], was found to strongly attenuate virus replication in IFN competent cells. This mutant virus was avirulent in vivo, and mice immunized with it were fully protected against challenge with wild‐type CHIKV [171]. A manipulation that slowed cleavage at the 1/2 site of RRV produced similar phenotypic effects [172]. These and other attenuated alphaviruses represent promising candidates for highly effective live attenuated vaccines to prevent infections of RRV, ONNV, MAYV and other alphaviruses pathogenic for humans.

In alphavirus‐infected cells, the accumulation of free nsP2 halts RC formation by cleaving P1234 polyprotein into P12 and P34. This mechanism is also employed by some of alphaviruses to establish superinfection exclusion (SIE), a phenomenon where a preexisting viral infection prevents secondary infection by the same or closely related virus [173, 174, 175, 176, 177, 178, 179, 180]. For instance, SINV infection inhibits replication of superinfecting SINV, SFV and RRV in mosquito (*Aedes albopictus*) cell lines [178]. Similarly, prior infection of *Aedes aegypti* mosquitoes with the insect‐specific Eilat virus delays the dissemination of CHIKV by 3 days [181]. The expression of nsP2 induces a phenotype that mimics SIE, offering a potential strategy for creating alphavirus‐refractory systems in mosquitoes [182, 183, 184]. As a proof of concept, Reitmayer and colleagues developed transgenic *Aedes aegypti* mosquitoes expressing nsP2 of CHIKV or SINV, demonstrating reduced replication and dissemination of the respective viruses in these mosquitoes [184]. This approach highlights the potential of developing nsP2‐expressing transgenic mosquitoes that restrict the dissemination of multiple pathogenic alphaviruses. Reduction of vector competence of a mosquito is, therefore, a promising arbovirus control strategy that can be applied to reduce cases of human infection as well as to limit the spread of alphaviruses to new geographical locations.

## 8 Perspectives

Significant progress has been made in identifying host factors as targets for treating alphavirus disease; however, targeting viral factors remains equally important [185, 186, 187, 188]. Targeting alphavirus‐encoded proteins, such as nsP2, with directly acting antivirals represents the most straightforward approach towards the development of specific treatments of diseases caused by alphaviruses. Despite numerous studies, many nsP2 functions remain poorly understood; furthermore, characterisation of nsP2 functions has been mostly performed using CHIKV, SFV, SINV, and, to a lesser extent, VEEV. Consequently, studies are needed to confirm whether the obtained findings apply equally across all alphaviruses. Moreover, many important activities of nsP2 are still unknown. In particular, there is a lack of information concerning the role of nsP2 in the early replicase complexes, including its interactions with viral genome and the exact role of its NTPase/RNA helicase activities. It is especially attractive to target the early stages of infection when the virus is not yet fully established and is most vulnerable. Further studies are needed to uncover these and other unknown facets of nsP2 in alphavirus infection.

As multi‐functional protein nsP2 is difficult to study and represents a challenging target. Furthermore, an individual nsP2 may not be the most promising target; instead, it could be nsP2 included to P1234 and P123 polyproteins, that are indispensable for alphavirus replicase complex formation. As of now, the development of drugs to prevent replicase complex formation is hampered by the lack of information about the structural organisation of P123 and P1234 polyproteins. The ability of nsP2 to process P1234 polyprotein into P123 and nsP4—a critical event for replicase complex formation—both in cis and *in trans* represents an additional hurdle. Studies of functions of nsP2 functions using advanced methods of structural biology, single molecule trafficking, etc, would offer new possibilities for targeting this protein with antiviral compounds. As of now, compared to targeting the protease activity of nsP2, targeting its other functions has been clearly under‐explored. Recent advances, especially resolving the structure of the core of alphavirus replicase, open perspective to apply rational drug development approaches to target helicase part of nsP2 and/or inhibit its interaction with nsP4. Such approaches could be combined with the development of high throughput screening compatible cell‐based assays using P1234 polyprotein. The combination of these assays holds a key for the identification of potent broad‐spectrum inhibitors that could serve as leads for the development of direct‐acting antivirals to treat alphavirus infections.

Increasing our understanding of nsP2's structure, functions, and interactions could also lead to broadly applicable strategies for alphavirus attenuation, supporting the development of novel live attenuated vaccines. It could be expected that these vaccines will, similar to IxChiq (the licenced vaccine against CHIKV), create long‐lasting protection. The combination of efficient vaccines with potent, directly acting antivirals represents a unique and powerful combination allowing new treatment/prevention strategies as well as unique opportunities to counter problems caused by rare side effects of live attenuated vaccines.

## Author Contributions

S.W. and A.M. conceptualised and draughted the manuscript. S.M. critically reviewed the manuscript. All authors approved the final version of the manuscript.

## Conflicts of Interest

Andres Merits is listed as an inventor on patent number 11201907272Y, titled ‘*Alphavirus nsP Mutants as Vaccines*’ (March 27, 2024). Suresh Mahalingam is an inventor on patent number WO/2017/201579, titled ‘*Arthrogenic Alphavirus Vaccine*’. Suresh Mahalingam serves as an editorial board member for *Reviews in Medical Virology*.

## Data Availability

The authors have nothing to report.
